# Towards sustainable maize production in the U.S. upper Midwest with interseeded cover crops

**DOI:** 10.1371/journal.pone.0231032

**Published:** 2020-04-09

**Authors:** Hannah L. Rusch, Jeffrey A. Coulter, Julie M. Grossman, Gregg A. Johnson, Paul M. Porter, Axel Garcia y Garcia

**Affiliations:** 1 Department of Agronomy and Plant Genetics, University of Minnesota, St. Paul, Minnesota, United States of America; 2 Department of Horticulture, University of Minnesota, St. Paul, Minnesota, United States of America; 3 Southern Research and Outreach Center, University of Minnesota, Waseca, Minnesota, United States of America; 4 Southwest Research and Outreach Center, University of Minnesota, Lamberton, Minnesota, United States of America; Michigan State University, UNITED STATES

## Abstract

The incorporation of cover crops into the maize (*Zea mays* L.)-soybean [*Glycine max* (L.) Merr.] rotation in the U.S. upper Midwest may improve sustainability. Long, cold winters in the region make identifying successful cover crop species and management practices a challenge. Two experiments were conducted in Minnesota, USA from fall 2016 through spring 2019 to examine the effect of cover crops interseeded at four- to six-leaf collar (early-interseeded) and dent to physiological maturity (late-interseeded) on biomass and grain yield of maize. Annual ryegrass (*Lolium multiflorum* L.) and cereal rye (*Secale cereale* L.) were evaluated as monocultures and in mixtures with crimson clover (*Trifolium incarnatum* L.) and forage radish (*Raphanus sativus* L.). Differences in canopy cover and biomass of late-interseeded cover crops were observed at the southernmost location in 2018. Additional accumulated growing-degree days in fall 2018 did not translate into increased cover crop canopy coverage of late-interseeded cover crops. Differences in cover crop canopy cover and biomass of early-interseeded cover crops were observed by fall frost at all locations in 2017 and at the northernmost location in 2018. Cover crop canopy cover and biomass at termination before planting maize, soil moisture at maize planting as well as maize aboveground biomass and yield were not affected by spring cereal rye regrowth of cover crops late-interseeded the previous year. Similarly, early-interseeded cover crops did not affect maize aboveground biomass or yield. We attribute these results to limited cover crop growth. This highlights the potential of a variety of cover crop strategies interseeded into maize in the U.S. upper Midwest; however, efforts to fine-tuning cover crop management and weather conditions are needed to benefit from such practice.

## Introduction

The maize (*Zea mays* L.)-soybean [*Glycine max* (L.) Merr.] rotation dominates agricultural production in the U.S. upper Midwest. This system is characterized by mechanization, high external inputs, and an extended fallow period. During the fallow period between harvest and planting, soils are vulnerable to erosion and essential plant nutrients can be lost to ground and surface waters. Incorporating cover crops into the maize-soybean rotation can help prevent these losses and thereby increase sustainability.

Cover crops deliver multiple ecosystem services [1], such as reduced nutrient leaching [2,3] through nutrient uptake [4], reduced soil erosion [5], enhanced soil fertility [6] and improved soil-water dynamics [7], weed suppression [8,9], and forage production [10]. Cover crops are promoted as a best management practice to avoid water quality impairment [11] and as a soil management tool [5], but their adoption remained low by the mid 2010s [12]. In northern climates, the period of time for cover crop establishment after the maize harvest in October or November is limited by available heat units and daylight hours. However, interseeding cover crops into maize before harvest may improve establishment and function.

Little is known about the potential for integrating cover crops into the maize-soybean rotation. In Minnesota, cover crops interseeded into maize at the seven-leaf collar stage reduced soil nitrate in spring, thus reducing the potential for nitrate leaching without reducing maize yield [13]. Cover crops did, however, reduce soil water content in a dry season and reduced soybean yield when they were not adequately terminated [13]. Another study in Minnesota found that cereal rye (*Secale cereale* L.; CR) aerially interseeded into maize or soybean in mid-August to mid-September produced more than 0.050 Mg ha^-1^ of biomass in 40% of the instances observed [14].

Until more is known about the consequences of interseeding cover crops, the practice is unlikely to be widely adopted by maize producers. Additionally, more information is needed on the viability of alternative cover crops for the region. Until recently, research on cover crops in the U.S upper Midwest focused on a few species. Cereal rye is among the most popular cover crops in the United States [15]. The literature on CR provides insight into the best timing for planting to maximize biomass [16], termination timing to avoid allelopathic effects [17], and establishment options [13,14,18].

This study aims to increase the knowledge of cover crop interseeding options for the U.S. upper Midwest. To this end, six cover crop strategies, including CR as well as underrepresented cover crop species, were early- and late-interseeded into maize. The objectives were to: 1) compare the establishment and growth of cover crops interseeded at four- to six-leaf collar and dent to physiological maturity maize stages of development across multiple environments, 2) evaluate the effect of interseeding CR into maize at dent to physiological maturity stages on regrowth in the springtime and on soil moisture at maize planting, and 3) assess the effect of interseeded cover crops on maize yield. The results of this study provide insight into possible outcomes of alternative cover cropping practices for maize-based cropping systems and additional management options.

## Materials and methods

### Experimental sites

Two field experiments were conducted from fall 2016 through spring 2019 at three Minnesota locations. Experiment 1 involved interseeding cover crops at maize dent to physiological maturity stages of development (hereafter referred as late-interseeded) and experiment 2 consisted of interseeding cover crops at four- to six-leaf collar stages of development (hereafter referred as early-interseeded). Both studies were conducted at the University of Minnesota Research and Outreach Centers in Grand Rapids (47°18’N, -93°53’W), Lamberton (44°24’N, -95°31’W), and Waseca (44°06’N, -93°53’W), Minnesota, USA. The late-interseeded study was conducted within the Minnesota Long-Term Agricultural Research Network (http://ltarn.cfans.umn.edu/). These three locations span a range of soil types, precipitation, and weather gradients. Soils were a well-drained Nashwauk loam (fine-loamy, mixed, superactive, frigid Oxyaquic Glossudalfs) at Grand Rapids, a moderately well drained Normania clay loam (fine-loamy, mixed, superactive, mesic Aquic Hapludolls) at Lamberton, and a somewhat poorly drained Nicollet clay loam (fine-loamy, mixed, superactive, mesic Aquic Hapludolls) at Waseca. Long-term (1990–2015) average annual cumulative precipitation is 700 mm in Grand Rapids, 708 mm in Lamberton, and 922 mm in Waseca; 75% of that precipitation falls during the growing season (April-September). For the same period, the long-term average annual maximum air temperature is 8°C in Grand Rapids and 13°C in Lamberton and Waseca. The long-term average annual minimum air temperature for the same period is -1°C in Grand Rapids, 1°C in Lamberton, and 2°C in Waseca.

### Experimental design

Both experiments were a randomized complete block design with four replications, except for the late-interseeded study in Grand Rapids, which had three replications. Plot size in the late-interseeded study was 3.0 m wide by 6.1 m long at all locations. Plot size in the early-interseeded study was 3.0 m wide by 9.1 m long at Grand Rapids, 3.0 m wide by 8.8 m long at Lamberton, and 4.6 m wide by 8.5 m long at Waseca.

Treatments included six cover crop strategies and a no cover crop control. Two grass species—annual ryegrass (*Lolium multiflorum* L.; AR) and CR—were used in a monoculture and in mixtures of two and three species. The two-species mixtures consisted of a grass plus crimson clover (*Trifolium incarnatum* L.; CC) and are denoted as ARCC and CRCC. The three-species mixtures included a grass, CC, and forage radish (*Raphanus sativus* L.; FR) and are denoted as ARCCFR and CRCCFR. The CR plots in the late-interseeded study were instrumented with ceramic cups to monitor NO_3_-N in the soil solution (not herein reported) and with access tubes to monitor soil moisture, which resulted in frequent visits. To avoid concerns with disturbance, a no cover crop control treatment was assigned to each grass species (Table 1) and are denoted as ARNC and CRNC. Cover crop species were selected based on functional traits (i.e., potential for N uptake and soil fertility improvement), phenological niche (i.e., winter hardiness), suitability for interseeding (i.e., shade tolerance), and seed availability. Cereal rye was the sole winter-hardy cover crop, while AR, CC, and FR winter-kill in this region.

**Table 1:** Cover crop seeding rates in Experiments 1 and 2.

Cover crop	Monoculture		2-species mixture		3-species mixture	
	AR	CR	ARCC	CRCC	ARCCFR	CRCCFR
	Seeding rate (kg ha^-1^)					
Annual ryegrass (AR)	28	-	14	-	14	-
Cereal rye (CR)	-	67	-	33.5	-	33.5
Crimson clover (CC)	-	-	22	22	16.5	16.5
Forage radish (FR)	-	-	-	-	10	10

### Agronomic management

Plots rotated each year between maize and soybean. During the experimental years all plots in the late- and early-interseeded studies received strip-tillage (15 cm deep, 20 cm wide) one to 15 d before planting maize in 76-cm wide rows at 86,000 seeds ha^-1^ at a depth of 5 cm with a 4-row planter (Table 2). For both studies, springtime regrowth of winter-hardy CR interseeded the previous year was terminated using 0.84 kg ae ha^-1^ of glyphosate [N-(phosphonomethyl)glycine] applied one to seven days before maize planting. Due to differences in growing season length, maize genotypes varied between locations. Maize in the late-interseeded study was a 76 RM hybrid (Pioneer P7632AM) at Grand Rapids and a 103 RM hybrid (DEKALB DKC53-56RIB) at Lamberton and Waseca. Maize in the early-interseeded study was a 76 RM hybrid (Pioneer P762AM1) at Grand Rapids, a 107 RM hybrid (Pioneer P0157AMX) at Lamberton, and a 99 RM hybrid (DEKALB DKC49-72RIB) at Waseca.

**Table 2:** Calendar of activities at late- and early-interseeded studies.

Activity	Grand Rapids			Lamberton			Waseca		
	2016	2017	2018	2016	2017	2018	2016	2017	2018
*Late-interseeded cover crops*									
Cover crop sampling	-	7-May	22-May	-	21-Apr	16-May	-	21-Apr	7-May
Cover crop termination	-	10-May	22-May	-	29-Apr	8-May	-	23-Apr	10-May
Maize planting	15-May	10-May	22-May	30-Apr	8-May	16-May	29-Apr	24-Apr	7-May
Cover crop interseeding	20-Sep	3-Sep	10-Aug	14-Sep	31-Aug	14-Aug	14-Sep	4-Sep	13-Aug
Maize harvest	25-Oct	26-Oct	13-Oct	17-Oct	25-Oct	26-Oct	16-Oct	30-Oct	16-Oct
Cover crop sampling	9-Nov	9-Nov	5-Nov	14-Nov	30-Oct	20-Oct	15-Nov	1-Nov	27-Oct
*Early-interseeded cover crops*									
Cover crop sampling	-	-	15-May	-	28-Apr	7-May	-	-	14-May
Cover crop termination	-	-	22-May	-	4-May	8-May	-	-	17-May
Maize planting	-	10-May	22-May	19-May	12-May	19-May	-	5-May	7-May
Cover crop interseeding	-	27-Jun	26-Jun	29-Jun	15-Jun	15-Jun	-	14-Jun	14-Jun
Maize harvest	-	9-Nov	5-Nov	21-Oct	24-Oct	18-Oct	-	29-Oct	29-Sep
Cover crop sampling	-	26-Oct	13-Oct	16-Oct	26-Oct	26-Oct	-	30-Oct	16-Oct

Nitrogen fertilizer in the late-interseeded study was broadcast applied at 73 kg ha^-1^ as urea (46-0-0, N-P-K) within one week of maize planting and sidedressed at 70 kg ha^-1^ as urea at the six-leaf collar stage of maize. Nitrogen and S fertilizers were applied in the early-interseeded study at Grand Rapids and Waseca at 63 kg N ha^-1^ as urea and 17 kg S ha^-1^ as gypsum (calcium sulfate dihydrate) within one week of maize planting, and an additional 101 kg N ha^-1^ as urea was sidedressed at the six-leaf collar stage of maize. In Lamberton, no fertilizer was applied at planting due to wet field conditions and 135 kg N ha^-1^ was sidedressed as urea at the six-leaf collar stage of maize. In 2017, fertilization at Lamberton was delayed such that cover crops in the early-interseeded study were interseeded before any fertilization was applied.

Weeds were controlled with a post-emergence herbicide approximately six weeks after maize planting. Weeds in the late-interseeded study were treated with glufosinate {(RS)-2-Amino-4-(hydroxy(methyl)phosphonoyl)butanoic acid} while glyphosate was applied in the early-interseeded study.

Cover crop seed was weighed by species in the lab and mixed at the field. Cover crops were manually broadcast in the early-interseeded study, corresponding to the time of maize sidedressing, and lightly incorporated with a rake in the late-interseeded study at all locations in 2017 and at Lamberton in 2018 as no rainfall was predicted.

Growing-degree days (GDD) for CR were calculated from 1 March through the first frost day (0°C minimum air temperature) in the fall using a minimum base air temperature of 4.4°C [19]. For maize, GDD were calculated from planting to harvest using a minimum base air temperature of 10°C. The maximum air temperature was set to 30°C for both CR and maize.

### Data collection

Cover crop canopy cover and biomass was measured in maize plots both in the fall when freezing air temperature remained consistent for three days (mid-October to early-November) and in the spring in maize stubble prior to termination of CR (late-April to mid-May). In most cases, fall canopy cover and biomass sampling occurred after maize harvest. However, in some cases, especially at Grand Rapids, freezing temperatures occurred before maize harvest and logistical constraints prevented harvesting maize before cover crop sampling in fall. A digital image was captured using the Canopeo app [20] to estimate the percentage of living green cover within a single 0.1-m^2^ quadrat per plot. Subsequently, all biomass within the quadrat was collected, placed in a brown paper bag, dried in a forced-air oven at 60°C until constant mass, and weighed.

Soil moisture was obtained on 7- to 10-d intervals at maize planting in late-interseeded cover crop plots with CR and the corresponding no cover crop control. A factory-calibrated PR2 soil moisture probe with an HH2 handheld readout device (Delta-T Devices, Cambridge, UK) was inserted into an access tube installed in the center of each plot to measure soil moisture as a percentage of volume at 0.10, 0.20, 0.30, 0.40, 0.60, and 1 m depths. The average of three measurements per depth was used as a final soil moisture value, and results from the top 30 cm of soil are presented in this study.

Three maize plants per plot were collected at physiological maturity. Maize was cut at 5 cm above the soil surface and ears were separated from stover. Stover was chopped in the field using a chipper. Maize stover and ears were dried in a forced-air oven at 60°C until constant mass and weighed. Maize grain weight and moisture content was measured after maize physiological maturity by harvesting the center two rows of each plot using a small-plot combine. Grain yield was calculated at 155 g kg^-1^ moisture.

### Statistical analysis

Data from each experiment were analyzed at P < 0.05 by analysis of variance with a linear mixed effects model (*lme4* package) [21] using R statistical software (R Core Team, 2013). Location, year, and cover crop strategy were considered fixed effects, and replication was considered a random effect. Soil moisture was analyzed at maize planting only, and depth was considered an additional fixed effect. Early-interseeded cover crop canopy cover and biomass at spring termination were analyzed separately by year due to no CR regrowth at Grand Rapids or Lamberton in 2019. When fixed effects were significant, means were compared with Tukey’s honestly significant difference test at P < 0.05 using the *lsmeans* package in R [22].

## Results

### Weather conditions

Compared to the long-term average, the 3-yr study period tended to be drier and warmer at Grand Rapids, but wetter and cooler at Lamberton and Waseca; the starting year 2016 was the wettest and warmest at all locations. Grand Rapids fall periods were wet, with rainfall ranging from 40 to 75 mm above the long-term average, and maximum and minimum temperatures ranging from 4.3 and 2.9°C above to -1.9 and -1.4°C below the long-term average, respectively. At Lamberton, the tendency towards wet and cool seasons was very clear, except for the 2016–2017 winter and 2018 spring, when rainfall was 25 and 6 mm below the long-term average, respectively. The 2018–2019 winter, with maximum air temperature at 5.6°C and minimum air temperature at 5.1°C below the long-term average, was by far the coldest season at Lamberton. At Waseca, the tendency towards wet and cool seasons was very clear as well; except for spring from 2016 to 2018, when it was drier than the long-term average (Table 3).

**Table 3:** Long-term (1990–2015) average weather conditions during the study period (2016–2019).

Season	Long-term weather^§^			Weather conditions during the study period (deviations from long-term)											
	Rainfall	Tmax	Tmin	Rainfall (mm)				Maximum Air Temperature (°C)				Minimum Air Temperature (°C)			
	(1990–2015)			2016	2017	2018	2019	2016	2017	2018	2019	2016	2017	2018	2019
Grand Rapids															
Fall	158	9.7	1.4	75	40	61		4.3	1.4	-1.9		2.9	0.0	-1.4	
Spring	186	8.5	-1.9	-82	-15	-52	-35	3.8	1.9	2.3	0.0	2.4	1.0	-0.5	-1.2
Summer	279	23.9	13.2	18	15	-42		0.9	0.2	1.1		0.4	-1.4	0.0	
Winter	77	-8.8	-16.7	81	-16	-39	-69	5.9	2.7	3.1	-0.2	6.1	1.6	-0.3	-3.8
*Year*	700	8.3	-1.0	92	23	-72		3.7	1.6	1.2		3.0	0.3	-0.6	
Lamberton															
Fall	161	15.1	2.1	91	44	103		1.9	0.1	-3.1		3.0	0.7	-0.8	
Spring	206	13.0	0.8	72	33	-6	127	2.1	-0.1	-2.5	-2.9	2.2	0.8	-0.5	-1.2
Summer	293	27.2	14.7	83	2	143		0.2	-0.5	-0.2		1.2	-0.1	1.7	
Winter	48	-2.8	-13.2	41	-25	28	9	0.9	2.4	-1.3	-5.6	2.8	3.0	-1.2	-5.1
*Year*	708	13.1	1.1	287	55	269		1.3	0.5	-1.8		2.3	1.1	-0.2	
Waseca															
Fall	203	14.5	2.9	194	-42	179		4.0	0.2	-2.8		3.6	0.4	-1.3	
Spring	260	12.6	1.7	-59	-20	-7	60	2.6	0.4	-1.5	-2.6	1.9	0.9	-1.0	-2.0
Summer	360	26.6	15.3	184	10	19		1.2	0.6	0.1		1.0	-0.3	0.7	
Winter	99	-3.5	-13.0	76	0	30	10	1.5	2.3	-1.1	-4.1	3.2	2.7	-1.2	-4.5
*Year*	922	12.6	1.7	396	-52	223		2.3	0.9	-1.3		2.4	0.9	-0.7	

^¶^ Spring = Mar–May, Summer = Jun–Aug, Fall = Sep–Nov, and Winter = Dec–Feb.

^§^ Rainfall = total seasonal rainfall (mm); Tmax = average maximum air temperature (°C), Tmin = average minimum air temperature.

**Fig 1:**
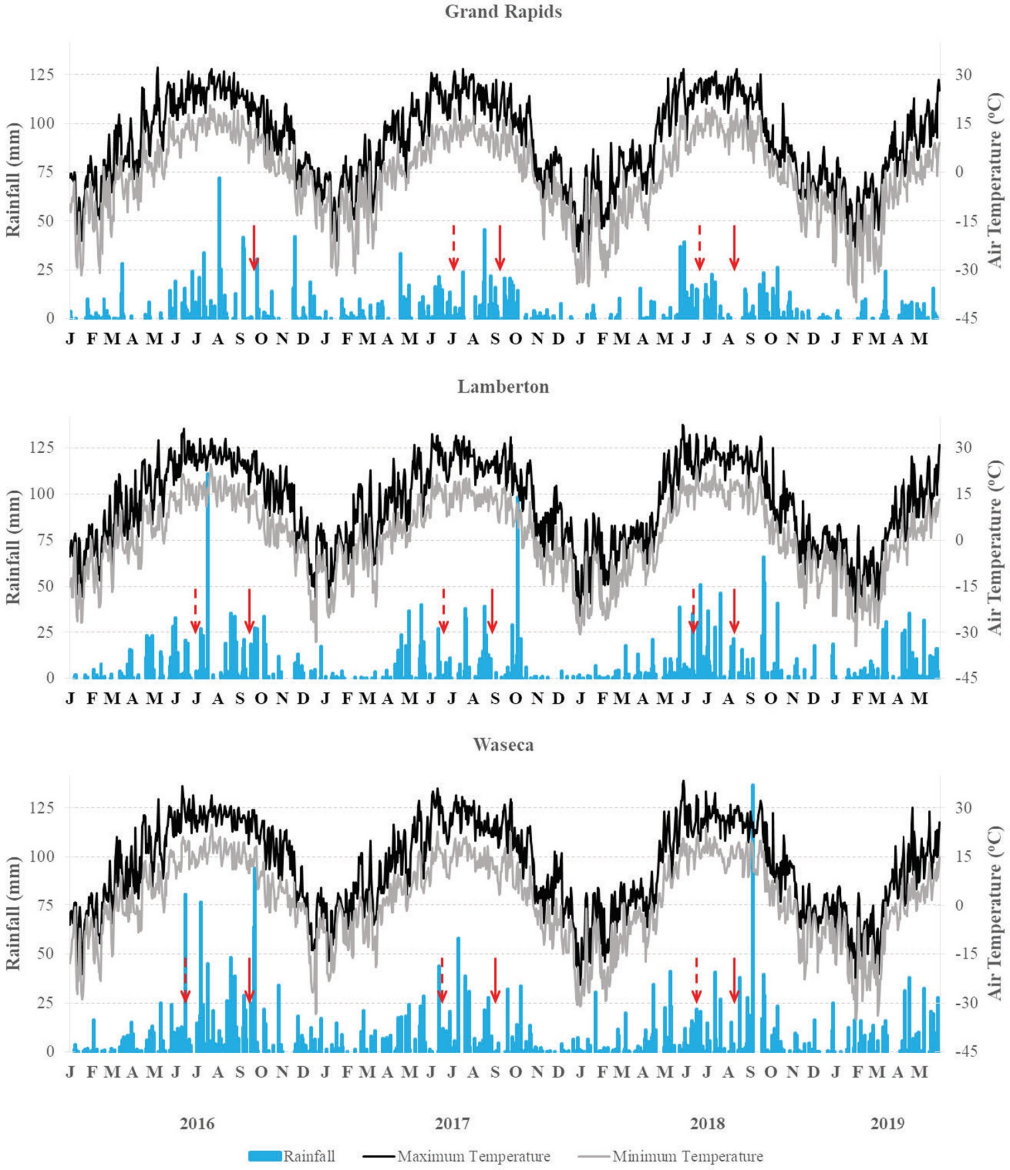
Weather conditions during the experimental years. Red arrows denote early- (dotted) and late-interseeded dates, approximately.

The early-interseeded cover crops study was conducted in 2017 and 2018 at Grand Rapids and from 2016 to 2018 in Lamberton and Waseca; due to flooding on 21–22 September 2016 (158 mm), the study at the Waseca site was abandoned. Early-interseeded cover crops at all locations received precipitation within one to two days of seeding. Cumulative precipitation seven days after seeding in 2016 was 9 mm in Lamberton while in 2017 and 2018 was 18 and 53 mm in Grand Rapids, 3 and 112 mm in Lamberton, and 15 and 80 mm in Waseca. The late-interseeded cover crops study was conducted from 2016 to 2018 at all locations. Late-interseeded cover crops at all locations received precipitation within five days of seeding in all years, except for Grand Rapids in 2018, which did not receive precipitation until 13 d after seeding. Cumulative precipitation seven days after seeding the late-interseeded study in 2016, 2017, and 2018 was 36, 4, and 0 mm in Grand Rapids; 19, 3, and 21 mm in Lamberton; and 89, 5, and 39 mm in Waseca (Fig 1).

Cover crop GDD accumulation varied among locations and years. At Grand Rapids, the early-interseeded cover crops accumulated 1300–1400 GDD from seeding to fall harvest, whereas at Lamberton and Waseca 400–500 more GDD were accumulated. Similarly, the late-interseeded cover crops at Grand Rapids accumulated fewer GDD compared with Lamberton and Waseca. Interseeding cover crops approximately two-weeks earlier in fall 2018 resulted in an additional accumulation of 181, 228, and 199 GDD before fall harvest at Grand Rapids, Lamberton, and Waseca, respectively (Table 4).

**Table 4:** Accumulated growing degree-days (GDD) of early- and late-interseeded cover crops at fall frost and before termination in the spring.

Location	Period	Cumulative Growing-Degree Days (GDD)^†^					
		Early-interseeded cover crops			Late-interseeded cover crops		
		Fall^¶^	Spring^§^	Full Season	Fall^¶^	Spring^§^	Full Season
Grand Rapids	2016–2017	-	-	-	-	-	-
	2017–2018	1375	217	1592	445	217	662
	2018–2019	1331	185	1516	627	187	814
	*Average*	*1353 (±31)*	*201 (±23)*	*1554 (±54)*	*536 (±129)*	*202 (±21)*	*738 (±107)*
Lamberton	2016–2017	1818	270	2088	528	277	805
	2017–2018	1824	210	2034	614	285	899
	2018–2019	1877	266	2143	725	296	1098
	*Average*	*1840 (±32)*	*249 (±34)*	*2088 (±55)*	*622 (±99)*	*286 (±10)*	*934 (±150)*
Waseca	2016–2017	-	-	-	605	217	822
	2017–2018	1870	268	2138	725	75	792
	2018–2019	1795	236	2031	762	248	998
	*Average*	*1833 (±53)*	*252 (±23)*	*2085 (±76)*	*697 (±26)*	*180 (±122)*	*871 (±146)*

^†^ Cumulative growing-degree days for cereal rye calculated from seeding using 4.44°C and 30°C as absolute minimum and maximum temperatures.

^¶^ For both early- and late-interseeded cover crops, GDD at fall were calculated from seeding to first day at 0°C average air temperature.

^§^ For both early- and late-interseeded cover crops, GDD at spring were calculated from fall of the first year of a given period to termination in the spring of the second year of that period.

### Canopy cover and biomass of late-interseeded cover crops

Canopy cover and biomass at fall frost of late-interseeded cover crops were affected by location, year, and strategy; only canopy cover was affected by the location x year interaction. At spring termination; however, canopy cover was affected by location, year, and by the location x year interaction while biomass was affected by year only (Table 5).

**Table 5:** Significance of fixed effects (P > F) for late-interseeded cover crop canopy cover and biomass at fall frost and spring termination, soil moisture at maize planting, and maize aboveground biomass and yield response to six cover crop strategies interseeded into maize at Grand Rapids, Lamberton, and Waseca, MN in 2016–2018.

Source of fixed variation†	Fall frost		Spring termination		Soil moisture at maize planting	Maize aboveground biomass	Maize yield at 15.5% moisture
	Cover crop canopy cover	Cover crop biomass	Cover crop canopy cover	Cover crop biomass			
Location (L)	<0.01	<0.01	<0.01	0.63	<0.01	<0.01	<0.01
Year (Y)	<0.01	<0.01	<0.01	<0.01	0.01	<0.01	0.03
Cover crop strategy (C)	<0.01	<0.01	0.23	0.15	0.14	0.76	0.63
Soil depth (D)	-	-	-	-	<0.01	-	-
L x Y	<0.01	0.24	<0.01	0.26	<0.01	<0.01	<0.01
L x C	0.05	0.11	0.954	0.26	0.12	0.37	0.94
Y x C	0.79	0.68	0.27	0.24	0.87	0.26	0.97
L x D	-	-	-	-	<0.01	-	-
Y x D	-	-	-	-	0.97	-	-
C x D	-	-	-	-	0.52	-	-
L x Y x C	0.59	0.82	0.93	0.16	0.90	0.84	0.29
L x Y x D	-	-	-	-	0.01	-	-
L x C x D	-	-	-	-	0.49	-	-
Y x C x D	-	-	-	-	0.90	-	-
L x Y x C x D	-	-	-	-	0.33	-	-

**Fig 2:**
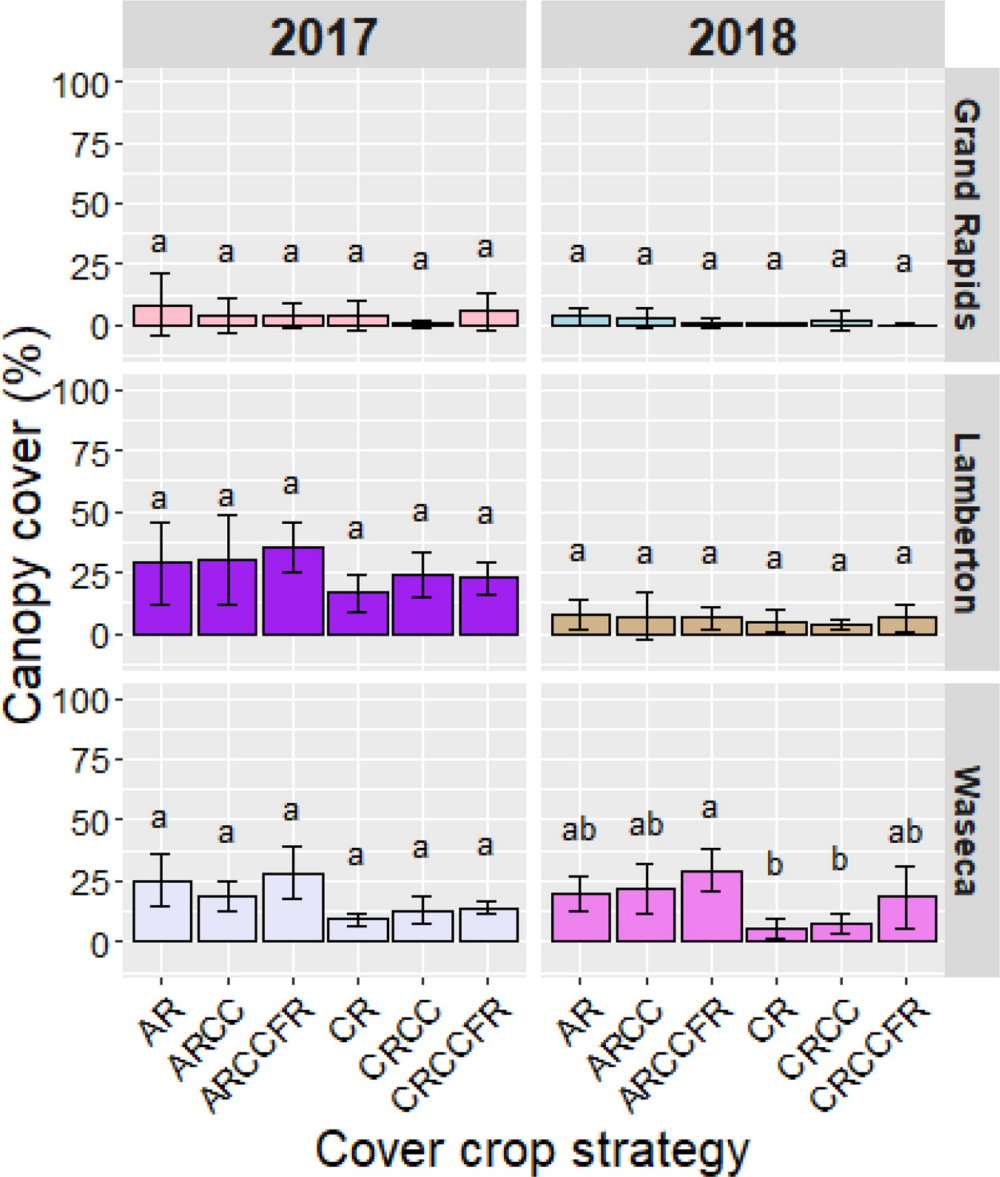
Canopy cover at fall frost of late-interseeded cover crops. For a given year within location, columns with different letters differ significantly at *P < 0.05*. Error bars are standard errors of the mean. AR = annual ryegrass, CC = crimson clover, FR = forage radish, CR = cereal rye.

Late-interseeded cover crops were seeded into maize at an earlier date in 2018, but greater GDD accumulation did not translate into more development or better canopy cover. At all locations and for all cover crop strategies, the average canopy cover in the fall was 35% or less in 2017 and 2018. Among locations, fall canopy cover of late-interseeded cover crops was least at Grand Rapids in both years, and greatest at Lamberton in 2017 and at Waseca in 2018. No differences in canopy cover were found among cover crop strategies either year of the study, except at Waseca in 2018 when ARCCFR produced significantly more canopy cover than CR or CRCC (Fig 2).

Mean fall biomass of late-interseeded cover crops at Grand Rapids was 0.076 Mg DM ha^-1^ in 2017 and significantly less (0.010 Mg DM ha^-1^) in 2018; at Lamberton was 0.149 and 0.076 Mg ha^-1^ in 2017 and 2018, respectively; and at Waseca, which showed the least year-to-year variation, was 0.158 and 0.134 Mg DM ha^-1^ in 2017 and 2018, respectively.

Mean spring (termination time) canopy cover and biomass of late-interseeded cover crops consisted of CR regrowth only and no differences were observed among cover crop strategies, despite the greater seeding rate of the CR monoculture versus the CRCC and CRCCFR mixtures. Canopy cover was significantly greater in 2017 than 2018 at Lamberton and Waseca for the pooled average of all cover crop strategies while biomass was greater in 2017 than 2018 at all locations (Table 6).

**Table 6:** Canopy cover and biomass at spring termination of late-interseeded cereal rye.

Location	Year	Canopy Cover (%)	Biomass (Mg DM ha^-1^)
Grand Rapids	2017	2.88±0.71 a^†^	0.34±0.06 a
	2018	0.63±0.31a	0.03±0.01 b
Lamberton	2017	44.63±3.82 a	0.49±0.06 a
	2018	2.62±0.59 b	0.01±0.00 b
Waseca	2017	18.27±2.38 a	0.43±0.08 a
	2018	1.98±0.54 b	0.03±0.00 b

^†^ Within a location in a column, means followed by different letters are significantly different at *P* < 0.05.

### Canopy cover and biomass of early-interseeded cover crops

Fall canopy cover and biomass of early-interseeded cover crops were affected by location, year, cover crop strategy, and their interactions except biomass, which was not affected by the location x year interaction. Canopy cover and biomass of CR at spring termination were not affected by year, cover crop strategy, and their interaction (Table 7).

**Table 7:** Significance of F values for fixed sources of variation for fall and spring canopy cover and biomass of early-interseeded cover crop as well as for aboveground biomass and grain yield of corn.

Source of fixed variation	Cover crop at fall frost^†^		Cover crop at spring termination^¶,§^		Maize^§^	
	canopy cover	biomass	canopy cover	biomass	biomass	grain yield
Location (L)	<0.01	<0.01			<0.01	<0.01
Year (Y)	<0.01	<0.01	0.054	0.065	0.0334	<0.01
Cover crop strategy (C)	<0.01	<0.01	0.214	0.600	0.977	0.198
L x Y	<0.01	0.195			<0.01	<0.01
L x C	<0.01	<0.01			0.702	0.351
Y x C	<0.01	<0.01	0.270	0.708	0.542	0.726
L x Y x C	<0.01	<0.01			0.439	0.0960

^†^ 2016, 2017, and 2018.

^¶^ No spring regrowth at Grand Rapids and Lamberton.

^§^ 2018 and 2019.

**Fig 3:**
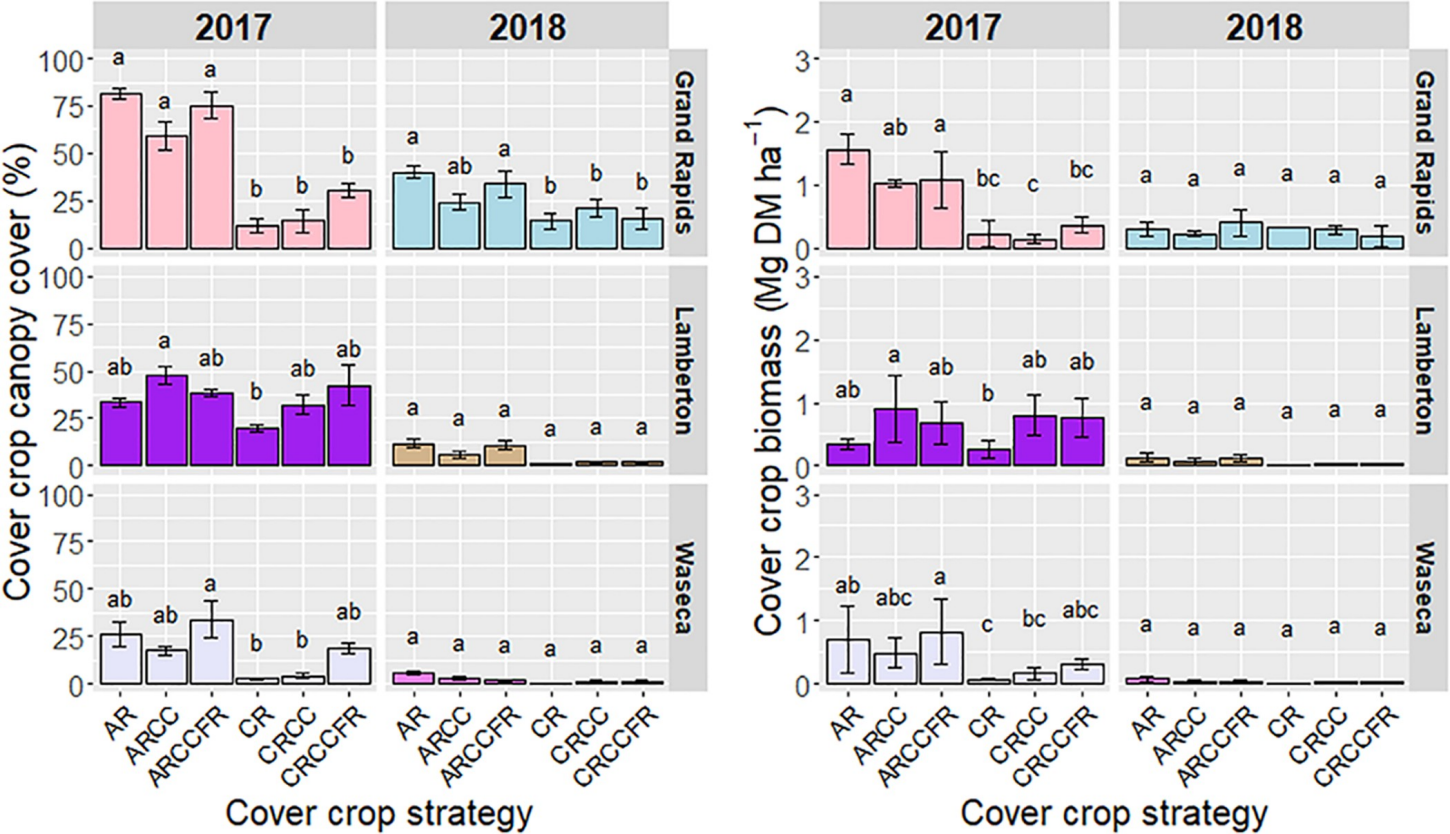
Canopy cover and biomass at fall frost of early-interseeded cover crops. For a given year within location, columns with different letters differ significantly at *P < 0.05*. Error bars are standard errors of the mean. Labels on the x-axis represent cover crop strategies: AR = annual ryegrass, CC = crimson clover, FR = forage radish, CR = cereal rye.

Fall canopy cover of early-interseeded cover crops varied widely within location and between years. At all locations, instances of canopy cover decreased from 2017 to 2018. In all locations and within a year, AR-based strategies produced greater canopy cover than CR-based strategies. At Grand Rapids, AR-based strategies had greater canopy cover than CR strategies in 2017 and 2018. At Lamberton, AR-based strategies had greater canopy cover than CR and CRCC in 2017, but no differences between cover crop strategies were observed in 2018. At Waseca, AR-based strategies tended to have greater canopy cover than CR-based strategies in both years; however, significant differences were observed only between ARCCFR and CR and CRCC in 2017 (Fig 3).

Fall biomass of early-interseeded cover crops ranged from 1.57 Mg DM ha^-1^ with AR at Grand Rapids in 2017 to 0 Mg DM ha^-1^ with CR at Waseca in 2018. At all three locations, AR-based strategies most frequently produced more biomass in the fall of 2017 compared to CR-based strategies. In fall 2017, CR and CRCC produced the least biomass at Grand Rapids and Waseca while both AR and CR produced the least at Lamberton. Fall biomass of cover crops in 2018 was marginal in all locations, with no differences among strategies at any location (Fig 3).

Cereal rye regrowth of early-interseeded cover crops in spring was low at all locations in 2018 and did not grow at Lamberton in spring 2019. Canopy cover was less than 2.5% and biomass did not exceed 0.035 Mg DM ha^-1^ at any location in 2018 or 2019. As a consequence, location, year, cover crops strategy, and interactions had no effect on spring canopy cover and biomass (Table 7).

### Effect of cover crops on soil moisture at maize planting

**Fig 4:**
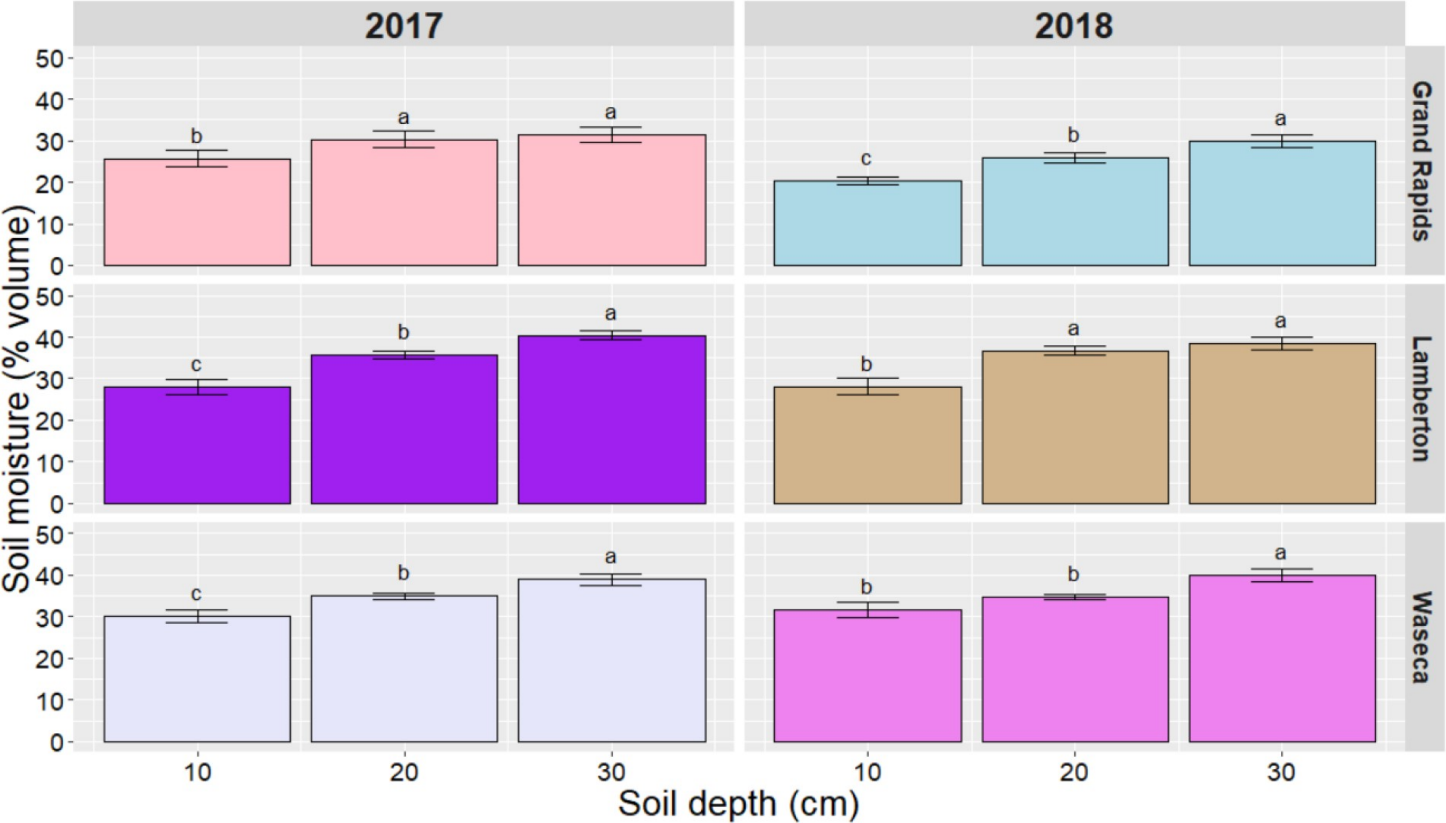
Mean soil moisture in the 0–10, 10–20, and 20–30 cm soil layers at maize planting after cereal rye cover crop termination in 2017 and 2018 at Grand Rapids, Lamberton, and Waseca. Different lowercase letters indicate means that are significantly different at *P < 0.05*. Error bars are standard errors of the mean.

Soil moisture was only monitored in the CR-based strategies of the late-interseeded study. The mean soil moisture from ground thaw until maize planting was used to determine differences between cover crop strategies. Our results showed that soil moisture at maize planting was not affected by cover crops, but it was affected by location, year, depth, and by the location x year and location x depth interactions (Table 5). Within a year, significant differences among soil layers were observed at all three locations. In all cases, the top 10 cm soil was drier than the 10–20 and 20–30 cm soil layers. The 0–10 cm soil layer had significantly (P < 0.05) less moisture than the other layers, except at Waseca in 2018 when no differences were observed between 0–10 and 10–20 (Fig 4).

### Effect of cover crops on biomass and grain yield of maize

Maize aboveground biomass at maturity and grain yield from the late-interseeded cover crops study were both affected by location, year, and by the location x year interaction, but were not affected by cover crop strategy (Table 5). At Grand Rapids in 2017, mean maize biomass (19.0 Mg DM ha^-1^) and grain yield (9.02 Mg ha^-1^) were less than in 2018 (22.1 Mg DM ha^-1^ and 9.95 Mg ha^-1^, respectively). Conversely, at Waseca in 2017, mean maize biomass (24.5 Mg DM ha^-1^) and grain yield (12.4 Mg ha^-1^) were greater than in 2018 (20.8 Mg DM ha^-1^ and 10.2 Mg ha^-1^, respectively). At Lamberton, biomass decreased from 25.6 Mg DM ha^-1^ in 2017 to 22.9 Mg DM ha^-1^ in 2018 while grain yield increased from 11.1 Mg ha^-1^ in 2017 to and 13.6 Mg ha^-1^ in 2018.

Similarly, aboveground biomass at maturity and grain yield of maize from the early-interseeded cover crops study were both affected by location, year, and by the location x year interaction, but no cover crop effect was observed (Table 7). Maize biomass at Grand Rapids was 26.7 Mg DM ha^-1^ in 2017 and 27.4 Mg DM ha^-1^ in 2018, at Waseca was 24.7 Mg DM ha^-1^ in 2017 and 22.7 DM Mg ha^-1^ in 2018, and at Lamberton was 18.2 Mg DM ha^-1^ in 2017 and 22.3 Mg DM ha^-1^ in 2018. Maize grain yield in 2017 and 2018 was 11.8 and 11.1 Mg ha^-1^, respectively at Grand Rapids; 11.1 and 13.6 Mg ha^-1^, respectively at Lamberton; and 11.6 and 9.19 Mg ha^-1^, respectively at Waseca.

## Discussion

### Factors affecting canopy cover and biomass of cover crop

We argue that in rainfed agriculture of northern climates weather conditions drive the success of cover crops use in conventional maize production systems. In this study, we interseeded cover crops early (four- to six-leaf collar stages) and late (dent to physiological maturity stages) in the maize growing season. At the northernmost location (Grand Rapids), low fall (2018) and spring (2019) biomass in the late-interseeded study was the result of poor establishment due to lack of water, as precipitation occurred 13 d after seeding cover crops. Our results support those from Wilson et al. [14] who reported lack of precipitation within seven days after air seeding as the factor limiting the establishment of cover crops in southeastern Minnesota. In contrast, at the southernmost locations of this study (Lamberton and Waseca), excess water negatively affected the establishment of early-interseeded cover crops; 238 mm of precipitation between 15 June and 14 July 2018 at Lamberton (~ half within one week of seeding) and 158 mm of precipitation on 21 September 2016 at Waseca, resulted in poor establishment and failure. Similarly, prolonged ponding at Waseca in August-September of 2018 led to limited canopy cover and low biomass of cover crops in the fall of that year and spring of 2019. Favorable conditions for CR growth in southwest Minnesota, defined as warmer than normal air temperature and near-average precipitation in fall and spring, have been characterized as occurring in 25% of the years [23]. Such favorable conditions occurred only during the 2016–2017 cover crop growing season in our study. Compared to the long-term averages, fall 2016 was wetter and warmer and the following spring varied from wetter and warmer at Grand Rapids to slightly drier and cooler at Waseca, favoring canopy cover and biomass of CR at spring termination in 2017. The wetter and colder than the long-term average conditions during the following fall and spring seasons along with late-spring snowfall in 2018 and 2019 contributed to the limited canopy cover and biomass of cover crops at spring termination. Wide variation in cover crop biomass is reported from previous studies in the region, with fall and spring biomass of early- and/or late-interseeded cover crops ranging from as little as 0.027 Mg DM ha^-1^ to as much as 2.13 Mg ha^-1^ [13, 14, 24]. Within a study, fall biomass of CR in southeastern Minnesota was reported at 0.027 Mg ha^-1^ in 2009 and 0.506 kg ha^-1^ in 2010 [14], nearly a 20-fold difference. In the present study, late-interseeded CR biomass decreased from 2017 to 2018 by approximately one-half at Waseca, two-fold at Lamberton, and nine-fold at Grand Rapids. Spring biomass of late-interseeded CR was less than 0.5 Mg ha^-1^ at all locations in 2017 and 2018, which is within or below the ranges reported from other studies in the region [14, 23, 25].

The cover crop species in this study are the most common choices for interseeding into maize and other crops in the U.S. [15], but interseeding comes with growth penalties associated with shade intolerance. In both of our studies, observed etiolated growth, and the low canopy cover and biomass obtained suggest that shade played a major role in the growth and development of the cover crops interseeded into maize. Similar results have been observed in studies interseeding cover crops at four- to six- and ten- to twelve-leaf collar stages in Ontario, Canada, reporting successful cover crops germination and establishment but stagnation and death under the maize canopy [26]. While the cover crops in our study did not die, the signs of stress from reduced light were evident. Of the four species we used, and in agreement with previous research results [27, 28], CC appeared to be tolerant to limited light; however, AR, CR, and FR are reported to be shade intolerant [28, 29, 30, 31, 32]. Our observations support previous research on interseeded legume cover crops into continuous irrigated maize reporting CC as more tolerant to shade than other legume species [28].

Our results were variable, but in agreement with survey reports indicating that stands of CR interseeding into maize production systems are highly variable [15]. Modeling studies for conditions in the central and upper U.S. Midwest [33] have also predicted penalties in CR biomass when interseeded into maize. This suggests the need for efforts to advance our understanding of the response of cover crops to shade, which in turn may open the opportunity for breeding efforts, as well as for a comprehensive characterization of cover crops potential for interseeding in maize production systems in the region.

AR-based strategies produced more canopy cover than CR-based strategies. Within the AR-based strategies, AR monoculture most often produced the greatest canopy cover and biomass, followed by ARCCFR and ARCC. Within the CR-based strategies, differences were not always significant, but the CR monoculture was most often the lowest producing for both early- and late-interseeded strategies. The latter suggests that a higher CR seeding rate did not result in greater canopy cover or biomass than other strategies. Previous research has shown that a higher CR seeding rate did not reduce N leaching any more than mixtures with lower seeding rates [34]. While CR may grow longer than AR in the fall due to its better capacity to withstand lower air temperatures, we hypothesize that AR performed better than CR because of its slightly better capacity to tolerate shade conditions [33], and because of a higher relative growth rate [35] compared to CR [36].

Cereal rye-based cover crop strategies did not result in differences in soil moisture at maize planting. This coincides with findings that mechanically terminated diverse cover crop mixtures did not reduce soil moisture [37]. Despite below-average precipitation at all locations in spring 2017 and 2018 (except Lamberton in 2017), cover crop strategies did not affect soil moisture in the 0–30 cm soil layer at maize planting compared with the CRNC treatment, which may be due to low springtime CR regrowth. A Minnesota study of soil moisture in a forage maize system with a CR cover crop showed that soil moisture after CR terminated between 25 to 28 April was similar to the control [17]. These results suggest that interseeding cover crops into maize in the region will have no effects in soil moisture.

### Cover crop effects on maize production

Early- and late-interseeded cover crops were not detrimental nor beneficial to maize biomass or grain yield. Our results are in agreement with those from a meta-analysis study [38] as well as those from field studies conducted in the region [13, 17, 18, 39], which found no effect of early- or late-interseeded grass cover crops on yield of maize. It has been reported, however, that cover crops may reduce maize yield when interseeded at two- to three-leaf collar stages [18], which is before our early-interseeded study. When yield penalties have been observed in maize with interseeded cover crops, weather [14, 18, 40] and management [13, 17, 41] have been reported as the major cause of yield reduction.

The low cover crop biomass observed in our study resulted in little to no competition between plant species and therefore had no effect on maize yield. Incidentally, studies reporting no effect of cover crops on yield of maize have also reported very low cover crops biomass. For example, spring biomass of CR interseeded in late season maize in Ontario, Canada, varied from 0.091 Mg ha^-1^ to 0.884 Mg ha^-1^ [41] while biomass of CR interseeded in early season maize in southern and southwest Minnesota, U.S. varied from 0.041 Mg ha^-1^ in the fall to near 1.000 Mg ha^-1^ in the following spring [13]; both studies report that CR cover crop had no effect on maize grain yield.

## Conclusion

This study provides new insight into the potential of cover crop monocultures and mixtures and their effect on maize productivity in the U.S. upper Midwest. It highlights the opportunity for broadcast interseeding cover crops early- (four- to six-leaf collar) and late- (dent to physiological maturity) in the maize growing season. Early- and late-interseeded annual ryegrass-based strategies produced greater total cover crop canopy cover and biomass by fall frost than cereal rye-based strategies in most cases. Our findings suggest that annual ryegrass may be an equally good or better option compared with cereal rye in terms of producing canopy cover and biomass as a cover crop. However, annual ryegrass winter kills, eliminating spring cover crop management before planting maize but also eliminating the opportunity to provide environmental services in the springtime. Increased GDD due to early seeding of late-interseeded cover crops did not translate into greater cover crop establishment in 2018. Conversely, early-interseeded cover crops accumulated more GDD thereby had greater development than late-interseeded cover crops in most cases. Our results show that early-interseeded cover crops produced highly variable results but was not detrimental to maize production. Regrowth of late-interseeded cereal rye did not reduce soil moisture at maize planting or subsequent maize biomass and grain yield. In both cases, we attribute these results to limited cover crop growth.

Additional research on the timing and methods of cover crop interseeding (e.g.: direct seeding), along with detailed information on corresponding field conditions, may lead to the identification of optimal interseeding times and potential tradeoffs of interseeding at different times during the growing season. Extending the study period or creating a controlled environment to observe the effects of soil moisture on maize yield might garner additional insight into the impact of spring cereal rye regrowth on maize productivity. Future research may seek to understand the impact of the cover crop strategies explored herein on soybean production to provide valuable information about their suitability and optimal placement within the maize-soybean rotation. Enhanced knowledge of how and when to best manage interseeded cover crops in maize cropping systems vis-à-vis our weather conditions may lead to greater soil cover and associated environmental, ecological, and management benefits during traditional fallow periods in the U.S. upper Midwest.
